# Tracing metabolic flux in vivo: motion pictures differ from snapshots

**DOI:** 10.1038/s12276-022-00842-9

**Published:** 2022-09-08

**Authors:** Il-Young Kim, Robert R. Wolfe

**Affiliations:** 1 Integrative Metabolic Fluxomics Lab, Department of Molecular Medicine, Lee Gil Ya Cancer and Diabetes Institute, Gachon University College of Medicine, Incheon, Korea; 2 Department of Geriatrics, Center for Translational Research in Aging & Longevity, Donald W. Reynolds Institute on Aging, University of Arkansas for Medical Sciences, Little Rock, AR USA

**Keywords:** Blood flow, Translational research

Since Rudolf Schoenheimer’s pioneering metabolic tracing work in the 1930s^1^, it is currently well appreciated that all constituents of living matter (e.g., DNAs, RNAs, proteins, lipids, and metabolites) are in a constant state of turnover at varying rates to achieve overall “dynamic” homeostasis. Furthermore, metabolic systems are highly complex, connected, and interactive, and consequently, one’s metabolic fluxes (“motion pictures”) should not be understood as individual components but as a whole system^2^. Unfortunately, most modern metabolic studies heavily depend on the measurements of static, snapshot information, so-called “statomics” (e.g., transcriptomics, proteomics, metabolomics, and cellular signaling cascades) of individual components of the whole system, which often fail to reflect actual metabolic status^3,4^. Without simultaneous considerations of metabolic flux, sole dependence on “statomics” can lead to incorrect conclusions regarding metabolic status. In this Special Feature, experts in the field of tracer methodology or fluxomics provide the basic principles and applications of the methodologies determining metabolic fluxes to various metabolic conditions. The incorporation of these state-of-the-art methodologies into metabolic research will guide researchers to a better understanding of dynamic metabolic systems with which to better dissect underlying molecular mechanisms of physiology or pathophysiology.

Currently, various tracer methodologies are employed to assess metabolic fluxes in vivo and in vitro, including rates of synthesis, breakdown, oxidation, conversion, and/or transport across the membrane^5,6^. While a great number of methods determining metabolic flux rates are currently used, any calculation of those metabolic fluxes (or kinetics) must be predicated on one of four tracer model structures or combinations: tracer dilution in (1) single-pool or (2) multiple-pools and tracer incorporation with (3) single precursor or (4) multiple precursors in steady or nonsteady states. In this Special Feature, Kim et al.^7^ discuss the importance of obtaining kinetic information on the in vivo metabolism of compounds and then the fundamental principles of tracer methodology and their use to assess in vivo metabolic flux rates. The authors focus on the basic model structures of tracer methodology that apply to a wide range of applications with emphasis on stable isotope tracers.

Dietary protein intake plays many physiological roles, particularly in the maintenance or gain of body protein stores, the largest storage site of which is skeletal muscle. Gains in muscle mass (i.e., hypertrophy) are accomplished through stimulation of net positive protein synthesis (i.e., rates of protein synthesis exceeding breakdown), called an anabolic response^8^. The recommended dietary allowance (RDA) for protein, defined as 0.8 g protein/kg/day, is in fact the minimal amount of protein intake that leads to net protein balance at the whole-body level, regardless of age, exercise training status, or clinical condition^7^. However, many physiological circumstances may require greater protein intake to maintain lean body mass (more importantly muscle mass). It is of critical importance to use an appropriate and accurate methodology to assess the optimal rate of dietary protein or amino acid intake. In this Special Feature, Wolfe et al.^9^ discuss stable isotope tracer methodology that enables a variety of approaches to assess the optimal dietary protein intake in humans. The authors primarily focus on tracer methods for estimating dietary protein and essential amino acid requirements for humans under different physiological conditions and discuss key methodological approaches, along with the advantages and limitations of each tracer method.

Once thought to be a dead-end waste product of anaerobic glycolysis due to the lack of oxygen, we now know that lactate is formed under fully aerobic conditions, and the formation stems from the imbalance between the rates of production and disposal (via oxidation and other pathways)^10^. Furthermore, lactate plays a number of key roles in metabolism, specifically being involved in intermediary metabolism (acting as a carbon energy carrier, myokine, and exerkine), redox biology, allosteric regulation of lipolysis, mitochondrial biogenesis and energetics, angiogenesis, gene expression (via histone lactylation), and brain metabolism^11^. In this Special Feature, Brooks et al.^12^ discuss the historical background related to the discovery of the lactate shuttle, concepts that describe the roles of lactate in the delivery of oxidative carbon energy and gluconeogenic precursors as well as in cellular signaling. These discoveries have been largely based on stable isotope tracer methodology.

We now know that various lipids are in constant states of rapid flux to achieve overall “dynamic” homeostasis in the body thanks to earlier studies using tracer methodology^1^. Over the past decades, there have been further advances in stable isotope tracer techniques and mass spectrometry that have advanced our current understanding of lipid metabolism, particularly in humans, regarding normal conditions and pathophysiology, such as obesity, fatty liver disease, and dyslipidemia. In this Special Feature, Salvador et al.^13^ discuss the advances in the quantitative approaches to determining lipid flux rates using stable isotope-labeled substrate tracers or ^2^H_2_O, including tracing flux rates of various fatty acid species and the entry of dietary fats and their fates (e.g., plasma and liver triacylglycerol turnover). The authors discuss how advances in fluxomics can be combined with recent advances in lipidomics to bring about further advancement in our understanding of lipid metabolism with an additional discussion on the potential use of data science (artificial intelligence and machine learning).

The combination of arteriovenous balance (A-V balance) techniques and (stable) isotope tracer methodologies enables quantification of metabolic kinetics of multiple metabolites across or between organs (i.e., metabolite exchanges) in steady state as well as nonsteady state (e.g., following a meal or during exercise)^14,15^. Described briefly by other contributors^7,9,12^ in this Special Feature, Bae et al.^16^ provide a more comprehensive overview regarding arteriovenous metabolite gradient measurement, including the historical perspectives on the development of A-V balance measurements, and discuss the advantages and limitations of the techniques with key examples of studies that have uncovered dynamic exchanges of numerous metabolites between organs in the contexts of various pathophysiological conditions.

Altogether, tracer methodology, particularly stable isotope tracers, can provide in-depth information on dynamic metabolic status (e.g., oxidation, synthesis, breakdown, transport, and exchanges), which can serve as a kinetic basis for the molecular mechanisms. The arteriovenous balance technique in conjunction with tracer methodology with advancements in powerful mass spectrometry enables the quantification of substrate kinetics across and between organs, with additional information on intracellular kinetics. Furthermore, multidisciplinary approaches, including multiomics and data science (e.g., artificial intelligence and machine learning), integrated into metabolic fluxomics can aid the advances in metabolic research that enables a better understanding of the metabolic status of living organisms and thus leads to the discovery and development of effective drugs against various pathologies.
