# Author Correction: Tracing metabolic flux in vivo: motion pictures differ from snapshots

**DOI:** 10.1038/s12276-022-00861-6

**Published:** 2022-09-26

**Authors:** Il-Young Kim, Robert R. Wolfe

**Affiliations:** 1 Integrative Metabolic Fluxomics Lab, Department of Molecular Medicine, Lee Gil Ya Cancer and Diabetes Institute, Gachon University College of Medicine, Incheon, Korea; 2 Department of Geriatrics, Center for Translational Research in Aging & Longevity, Donald W. Reynolds Institute on Aging, University of Arkansas for Medical Sciences, Little Rock, AR USA

**Keywords:** Blood flow, Translational research

Correction to: *Experimental & Molecular Medicine* 10.1038/s12276-022-00842-9, published online 08 September 2022

After online publication of this article, the authors noticed an error in the REFERENCES section.

The correct statement of this article should have read as below.

“7. Kim, I.-Y., Park, S., Kim, Y., Kim, H.-J. & Wolfe, R.R. Tracing metabolic flux in vivo: basic model structures of tracer methodology. *Exp. Mol. Med*. 10.1038/s12276-022-00814-z (2022).

9. Wolfe, R.R., Kim, I.-Y., Park, S. & Ferrando, A. Tracing metabolic flux to assess optimal dietary protein and amino acid consumption. *Exp. Mol. Med*. 10.1038/s12276-022-00817-w (2022).

12. Brooks, G. A. et al. Tracing the lactate shuttle to the mitochondrial reticulum. *Exp. Mol. Med*. 10.1038/s12276-022-00802-3 (2022).

13. Salvador, A.F., Shyu, C.-R. & Parks, E.J. Measurement of lipid flux to advance translational research: evolution of classic methods to the future of precision health. *Exp. Mol. Med*. 10.1038/s12276-022-00838-5 (2022).

16. Bae, H., Lam, K. & Jang, C. Metabolic flux between organs measured by arteriovenous metabolite gradients. *Exp. Mol. Med*. 10.1038/s12276-022-00803-2 (2022).”

The authors apologize for any inconvenience caused.

The original article has been corrected.

